# Effect of Exposure to Second-Hand Smoke on the Quality of Life: A Nationwide Population-Based Study from South Korea

**DOI:** 10.1371/journal.pone.0138731

**Published:** 2015-09-22

**Authors:** Yeon Wook Kim, Chang-Hoon Lee, Young Sik Park, Yu-Il Kim, Chul Min Ahn, Ju-Ock Kim, Joo Hun Park, Sang Haak Lee, Jae Yeol Kim, Eun Mi Chun, Tae Hoon Jung, Kwang Ha Yoo

**Affiliations:** 1 Division of Pulmonary and Critical Care Medicine, Department of Internal Medicine, Seoul National University College of Medicine, Seoul, Republic of Korea; 2 Department of Internal Medicine, Chonnam National University Hospital, Gwangju, Republic of Korea; 3 Department of Internal Medicine, Yonsei University College of Medicine, Seoul, Republic of Korea; 4 Department of Internal Medicine, Chungnam National University School of Medicine, Daejeon, Republic of Korea; 5 Department of Internal Medicine, Ajou University School of Medicine, Suwon, Republic of Korea; 6 Department of Internal Medicine, The Catholic University of Korea, College of Medicine, Seoul, Republic of Korea; 7 Department of Internal Medicine, Chung-Ang University College of Medicine, Seoul, Republic of Korea; 8 Department of Internal Medicine, Ewha Womans University School of Medicine, Seoul, Republic of Korea; 9 Department of Internal Medicine, Chilgok Kyungpook National University School of Medicine, Daegu, Republic of Korea; 10 Department of Internal Medicine, Konkuk University College of Medicine, Seoul, Republic of Korea; School of Public Health of University of São Paulo, BRAZIL

## Abstract

**Background:**

Although exposure to second-hand smoke (SHS) has been associated with various medical conditions, only limited data are available on its association with health-related quality of life (HRQOL), particularly data obtained with the EQ-5D or EQ visual analogue scale (VAS).

**Methods:**

This cross-sectional study evaluated 10,532 adult never-smokers who participated in the fifth Korea National Health and Nutrition Examination Survey. By using linear regression models to adjust for possible confounders and incorporating survey weights in analyses, the association between exposure to SHS and HRQOL—measured with the EQ-5D index and the EQ-VAS score—was evaluated. Data were further stratified by the amount of exposure time.

**Results:**

After weighted analysis and adjustment, exposure to SHS was significantly associated with lower measures on the EQ-5D index (β = −0.007, *P* = 0.005) and EQ-VAS score (β = −1.936, *P* < 0.001). When comparing the unexposed group with the groups exposed <2h/day and ≥2h/day, exposure to a longer duration of SHS was significantly associated with lower scores on the EQ-5D index and the EQ-VAS score.

**Conclusion:**

In conclusion, exposure to SHS was associated with reduced HRQOL measured by the EQ-5D index and EQ-VAS score, revealing a dose-response relationship.

## Introduction

Tobacco smoking is well known for its harmful effects on general health owing to the increased risk of developing fatal diseases. For this reason, global public health efforts to decrease smoking rates have become a major challenge [1]. More than 5,000,000 deaths a year are estimated to be the direct result of tobacco use, and more than 600,000 deaths are the result of exposure to second-hand smoke (SHS) [2]. Epidemiological studies have reported the increased risk of cardiovascular disease, stroke, lung cancer, chronic respiratory symptoms, and impaired pulmonary function among individuals exposed to SHS, demonstrating that exposure to SHS, like active smoking, is one of the leading causes of preventable poor health in the developed world [3–6].

Despite its rapid economic development and its efforts to control smoking, South Korea still has a considerable smoking rate of 42.1% reported among adult men (≥19 years old) and 6.2% among adult women. In addition, 57.2% of men and 38.7% of women belonging to the never-smoker adult population were reported to have been exposed to SHS at the workplace in 2013 [7, 8]. Because it is known that active smoking leads to impairment of health-related quality of life (HRQOL), vulnerabilities due to decreased HRQOL and the potential development of mental health problems among never-smokers exposed to SHS should also be concerned [9].

HRQOL is considered an important tool to assess the physical, social, and mental functioning of individuals. Decreased HRQOL is known to be associated with increased risk of death, hospitalization, and increased health care costs at the population level [10, 11]. Although decreased HRQOL in current active smokers has been widely investigated, only limited data are available on the effects of exposure to SHS on HRQOL among never-smokers [9, 12, 13]. In addition, most of the previous studies on the association between smoking and HRQOL used the 36-item Short Form Health Survey (SF-36) to measure HRQOL [12–14]. However, in contrast to the EQ-5D index, instruments such as SF-36 have limited use because they do not include preference-based measures in their scoring algorithms [15].

The aim of this study was to evaluate the association between self-reported exposure to SHS and HRQOL among never-smokers in the general population using data obtained from a nationwide survey from South Korea: the fifth Korea National Health and Nutrition Examination Survey (KNHANES V), and using the EQ-5D index and EQ-VAS score, which are the most popular preference-based tools used to assess HRQOL to date [8, 16, 17].

## Materials and Methods

### Study population

The KNHANES is an ongoing surveillance system since 1998 to assess the health and nutritional status of the general population of South Korea. It is composed of three component surveys: health interview, health examination, and nutritional survey [8]. KNHANES V was conducted between 2010 and 2012 and collected data from 11,400 households in 576 randomly selected survey areas, which were drawn from the annual population census and resident registration population of South Korea [18]. The cohort included 25,534 participants. Trained interviewers administered questionnaires addressing various health-related information, including living and working environments, comorbidities, quality of life, and smoking habits as well as the degree of exposure to SHS. To avoid confounding by active smoking, the analysis in the present study was based on 10,532 never-smoker adults aged above 20 years, who had responded to the questionnaire used to measure the EQ-5D index and EQ-VAS score. The design of this study was approved and given an exemption from deliberation by the Institutional Review Board of Seoul National University Hospital. Since this study used data collected from KNHANES V, the institutional review board waived the need for written informed consent from the participants.

### Measure of SHS exposure

Whether a participant had exposure to SHS and data on the duration of exposure (in hours per day) were collected by assessing the responses to the prepared questionnaire. The questions “If you are regularly exposed to cigarette smoke at your workplace, how many hours per day are you exposed?” and “If there is an active smoker in your family, how many hours per day are you exposed to smoking at home?” were asked to each participant. Respondents could answer ‘unexposed’ if they were not exposed to SHS in the workplace or at home, or they could express the duration of exposure in hours per day if they were exposed to SHS for more than 1 hour per day. Because exposure to SHS of less than 1 hour per day was anchored to ‘0’ hours in each question, the database could not provide accurate values when added up for total exposure time. Therefore, to evaluate whether a dose-dependent relationship exists in association with HRQOL, participants were categorized into three groups prior to all analyses according to the total duration of daily exposure to SHS in the workplace or at home: unexposed, <2 hours per day, and ≥2 hours per day.

### Measure of HRQOL

The Korean versions of the EQ-5D index and EQ-VAS, approved by the EuroQoL Group, were used to measure the HRQOL of the participants. The EQ-5D descriptive system comprises five dimensions: mobility, self-care, usual activities, pain/discomfort, and anxiety/depression. Participants were asked to indicate their health status by choosing 1 of 3 levels: no problems, some problems, extreme problems. Elicited utility scores from each dimension were converted into an EQ-5D index using the mapping system provided by the EuroQoL and the quality weight calculation formula proposed in previous studies [19–21]. The EQ-5D index was anchored at 0 for very poor health and 1 for perfect health, indicating a preference for specific health status. Respondents were also asked to mark their self-rated health on a vertical, visual analogue scale (EQ-VAS). Endpoints were labeled ‘best imaginable health state’ (numbered 100) and ‘worst imaginable health state’ (numbered 0) [19].

### Statistical analysis

Analyses included comparisons between the groups unexposed and exposed to SHS. To produce estimates representative of the general population of South Korea, survey weights derived from the calculation of the post-stratification estimator and from adjustments in the non-response rate, and extreme weights were used in all analyses [18]. Continuous variables were described as means and standard errors and were compared using independent *t*-tests. Categorical variables were described as frequencies, and the χ^2^ test with Rao-Scott correction was used to make comparisons between the groups. Univariate linear regression analyses were performed to allow adjustment for other potential confounders, which were measured from the survey, and multivariate regression models with weight analysis were used to investigate whether exposure to SHS was independently associated with changes in HRQOL. The following potential confounding variables derived from the questionnaire and health examination were considered: age, sex, body mass index (BMI), employed status, individual economic status, self-reported any alcohol use, marital status, and comorbid conditions (diabetes mellitus, hypertension, depression, stroke, osteoarthritis, and asthma) which were self-reported if a participant had told by a doctor whether they have been diagnosed with the disease. Since the presence of one comorbid condition showed collinearities with other comorbid conditions, presence of any comorbid condition was introduced as a single variable. If the *P*-value in univariate analysis was less than 0.10, it was included in the multivariate model, and all variables were entered simultaneously. The significance of variables was assessed by the *P*-value, and the goodness of fitting of the model was evaluated by R-squared.

To evaluate the association in each EQ-5D dimension, polynomial regression models were used to assign the exposure groups to categorical variables and problem levels to outcome variables, assuming ‘no problems’ as level 1 and ‘extreme problems’ as level 3. To evaluate the association with converted EQ-5D index and EQ-VAS score, models were used to assign the obtained scores to outcome variables. In addition, means of the EQ-5D index and EQ-VAS score adjusted for variables included in the multivariate model were calculated. *P*-values of <0.05 were considered statistically significant. All analyses were performed using the STATA software, version 12.0 (StataCorp, College Station, Texas, USA).

## Results

### Baseline characteristics

**Fig 1:**
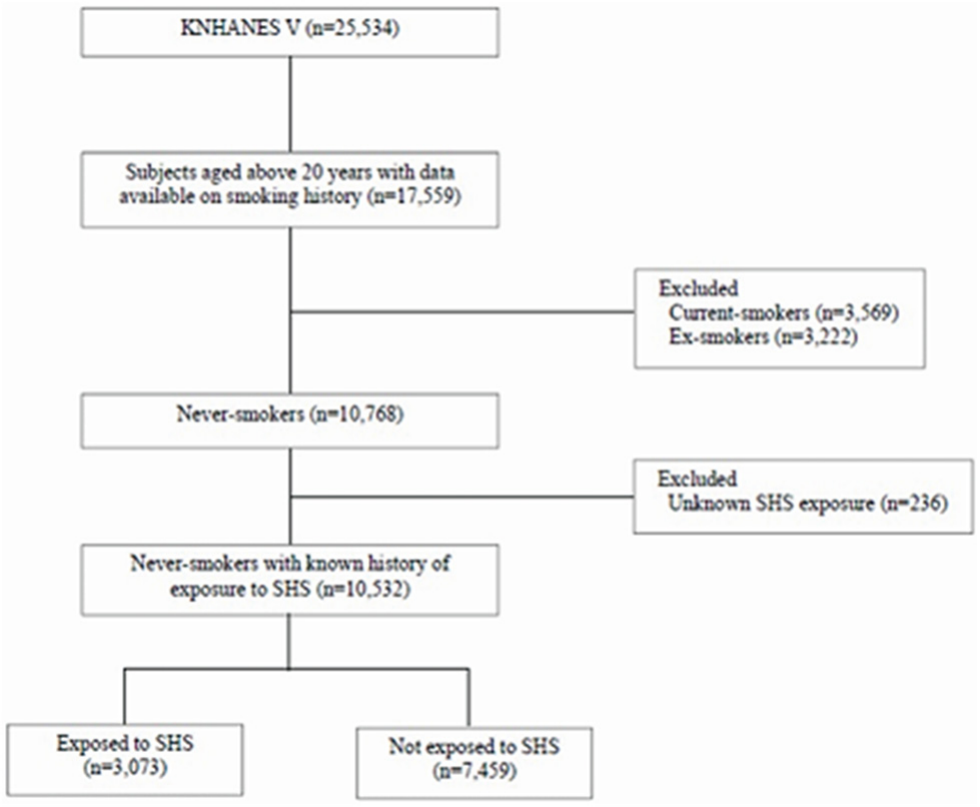
Flow Diagram of the Study Population.

Of the 17,559 adult respondents with data available on smoking history, 10,532 never-smokers had available exposure status to SHS and were eligible for analysis. Among the respondents included, 3,073 (29.2%) reported routine exposure to SHS, and 667 (6.3%) reported exposure of ≥2 hours per day (Fig 1). The comparison of baseline demographic data and clinical characteristics between the unexposed and exposed group are shown in Table 1. For the total estimated general population, the proportion of unexposed and exposed participants was 65.1% and 34.9%, respectively. Participants with exposure to SHS were generally younger, men, single, employed, and had received higher education. The proportion of participants with comorbid diabetes mellitus, hypertension, stroke, and osteoarthritis were higher in the unexposed group. Before any adjustments, calculated EQ-5D index and EQ-VAS score were higher in the exposed group.

**Table 1:** Demographic Characteristics of the Never-Smoker Population Estimated Using Survey Weight Analysis.

Characteristics	Unexposed to SHS	Exposed to SHS	*P*-value
Proportion of participants (%)	(65.1)	(34.9)	
Age (years), mean ± SE	48.3 ± 0.3	41.9 ± 0.3	<0.001
Sex, % of women	83.4%	72.7%	<0.001
BMI (kg/m^2^), mean ± SE	23.5 ± 0.1	23.5 ± 0.1	0.916
Comorbid condition, (%)			
Diabetes mellitus	14.0	9.8	<0.001
Hypertension	28.6	21.6	<0.001
Depression	4.8	4.3	0.103
Stroke	1.2	0.6	0.019
Osteoarthritis	13.4	8.4	<0.001
Asthma	3.0	3.1	0.725
Employed, (%)	44.0	81.2	<0.001
Any alcohol use, (%)	62.7	78.2	<0.001
Married, (%)	83.7	73.3	<0.001
Education, (%)			<0.001
Elementary school	27.0	15.0	
Middle school	9.2	9.9	
High school	32.0	40.5	
Higher education	31.9	34.7	
Personal economic status, (%)			0.593
1Q	26.1	25.1	
2Q	25.9	24.3	
3Q	25.3	25.4	
4Q	22.7	25.1	
EQ-5D index, mean ± SE	0.933 ± 0.002	0.956 ± 0.002	<0.001
EQ-VAS score, mean ± SE	73.755 ± 0.278	74.288 ± 0.393	0.019

SHS: second-hand smoke, BMI: body mass index

### Effect of exposure to SHS on EQ-5D and EQ-VAS

**Fig 2:**
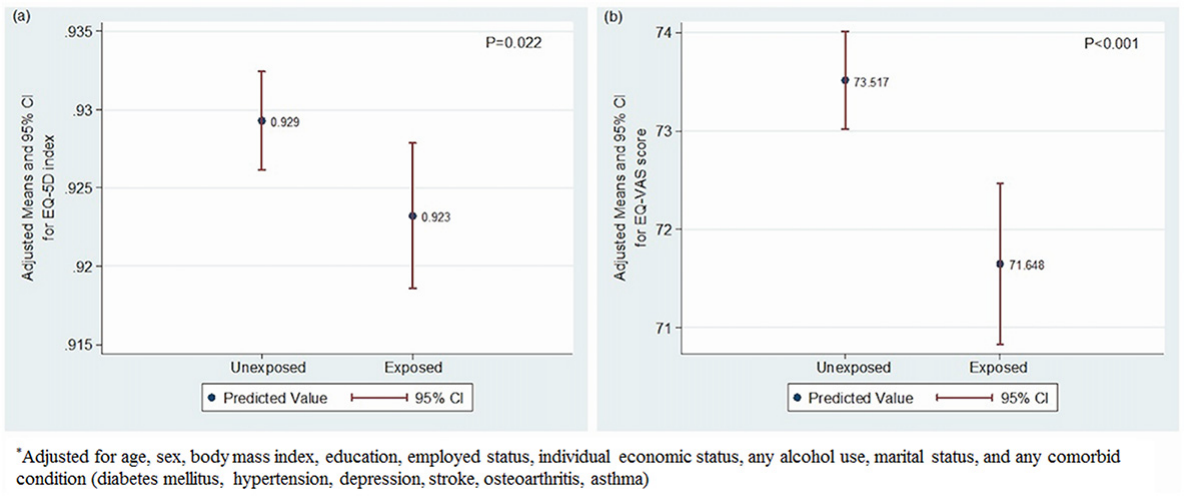
Adjusted Means of EQ-5D Index (A) and EQ-VAS Score (B) in the SHS-Exposed Group and the Unexposed Group.*

In univariate analysis, various clinical factors such as increasing age, sex, BMI, educational and economic status, any alcohol use, employed status, marriage, and presence of any comorbid condition showed strong effects on HRQOL. Multivariate regression models analyzing the association between HRQOL measures and exposure to SHS adjusted for pertinent confounders showed that after adjustment, exposure to SHS was an independent predictor of lower EQ-5D index (β = −0.007, *P* = 0.005, R^2^ = 0.232) and EQ-VAS score (β = −1.936, *P* < 0.001, R^2^ = 0.093) (S1 Table and Table 2). Although all the parameters did not reach statistical significance, a trend of higher problem levels in most of the dimensions of EQ-5D were found in the exposed group. Compared to the unexposed group, the group exposed to SHS had significantly lower adjusted means of EQ-5D index (0.929 *vs*. 0.923, *P* = 0.022) and EQ-VAS score (73.517 *vs*. 71.648, *P* < 0.001) (Fig 2). When further stratified by sex, adjustments in the EQ-5D index and EQ-VAS score showed that exposure to SHS was significantly associated with lower EQ-5D index (β = -0.008, *P* = 0.014, R^2^ = 0.226) and EQ-VAS score (β = -2.138, *P*<0.001, R^2^ = 0.084) in the women population. Though not reaching statistical significance, similar trend of association was also shown in men (S2 Table).

**Table 2:** Adjusted Differences in HRQOL Measures in the SHS-Exposure Group Compared with the Unexposed Group.*

HRQOL measures	Variable Estimate (SE)	95% CI	*P*-value
EQ-5D dimension^†^			
Mobility	0.127 (0.091)	-0.052–0.305	0.162
Self-care	-0.013 (0.162)	-0.331–0.304	0.934
Usual activities	0.087 (0.111)	-0.130–0.304	0.430
Pain/discomfort	0.104 (0.074)	-0.042–0.250	0.161
Anxiety/depression	0.134 (0.088)	-0.039–0.307	0.129
EQ-5D index	-0.007 (0.003)	-0.013 –-0.002	0.005
EQ-VAS score	-1.936 (0.455)	-2.830 –-1.042	<0.001

HRQOL: health-related quality of life, SHS: second-hand smoke, CI: confidence interval.

*Adjusted for age, sex, body mass index, education, employed status, individual economic status, any alcohol use, marital status, and any comorbid condition (diabetes mellitus, hypertension, depression, stroke, osteoarthritis, asthma).

† Calculated using polynomial ordinal logistic regression analysis, evaluating the variable estimate (ß coefficient) for higher problem level in each dimension.

Table 3 provides the adjusted problem levels of each EQ-5D dimension, the EQ-5D index, and the EQ-VAS score in comparison between the groups with daily exposure of <2 h/day and ≥2 h/day with the unexposed group. Compared to the unexposed group, groups with longer daily exposure to SHS trended to have higher problem levels in all 5 dimensions of EQ-5D. Adjustments in the EQ-5D index and EQ-VAS score in the comparison between the groups showed that longer daily SHS exposure of ≥2 h/day was significantly associated with lower EQ-5D index (β = −0.011, *P* = 0.013) and EQ-VAS score (β = −3.631, *P* < 0.001). The association revealed a dose-response relationship.

**Table 3:** Adjusted Differences in HRQOL Measures in the Groups with Exposure to SHS of <2 h/day and ≥2 h/day Compared with the Unexposed Group.*

HRQOL measures	Variable Estimate (SE)	95% CI	*P*-value
EQ-5D dimension^†^			
Mobility			
<2 h/day	0.082 (0.101)	-0.116–0.280	0.416
≥2 h/day	0.256 (0.158)	-0.055–0.566	0.106
Self-care			
<2 h/day	-0.206 (0.184)	-0.568–0.156	0.264
≥2 h/day	0.468 (0.272)	-0.066–1.003	0.086
Usual activities			
<2 h/day	-0.003 (0.125)	-0.249–0.244	0.983
≥2 h/day	0.341 (0.178)	-0.009–0.691	0.056
Pain/discomfort			
<2 h/day	0.053 (0.083)	-0.111–0.216	0.527
≥2 h/day	0.266 (0.115)	0.040–0.491	0.021
Anxiety/depression			
<2 h/day	0.093 (0.096)	-0.095–0.282	0.330
≥2 h/day	0.264 (0.153)	-0.037–0.565	0.085
EQ-5D index			
<2 h/day	-0.006 (0.003)	-0.012 –-0.001	0.023
≥2 h/day	-0.011 (0.004)	-0.020 –-0.002	0.013
EQ-VAS score			
<2 h/day	-1.468 (0.491)	-2.433–-0.503	0.003
≥2 h/day	-3.631 (0.814)	-5.229–-2.033	<0.001

HRQOL: health-related quality of life. SHS: second-hand smoke, CI: confidence interval.

*Adjusted for age, sex, body mass index, education, employed status, individual economic status, any alcohol use, marital status, and any comorbid condition (diabetes mellitus, hypertension, depression, stroke, osteoarthritis, asthma).

† Calculated using polynomial ordinal logistic regression analysis, evaluating the variable estimate (ß coefficient) for higher problem level in each dimension.

## Discussion

The present study, which included a large nationwide cohort from South Korea, indicates that exposure to SHS is significantly associated with lower HRQOL, measured by using both the EQ-5D index and EQ-VAS score, in a dose-response manner. These findings are important not only because HRQOL is a major outcome closely related with hospitalization and mortality but also because HRQOL is an important indicator for estimating the level of public health and establishing health-related policies [10, 22]. Our study provides novel data and demonstrates the association between exposure to SHS and HRQOL in the never-smoker population using reliable methods to measure these variables, that is, a questionnaire to obtain data of exposure to SHS, and EQ-5D index/EQ-VAS score to measure HRQOL, which is the most popular preference-based tool used to assess HRQOL to date [16, 17].

A few studies have evaluated the effects of SHS on HRQOL and reported decreased HRQOL scores in participants exposed to SHS. However, most of these studies evaluated the association between exposure to SHS and HRQOL in patients with underlying pulmonary disease or heart problems, and the limited data corroborating this association in the general population underscore the importance of our results [12, 13, 23]. Moreover, in most of the previous studies, the scoring system used for evaluating HRQOL was SF-36, a non-preference-based measure form [15]. Considering that the use of cost-utility analysis has been recently emphasized to compare the beneficial effects of a healthcare program, efforts have been focused on comparing and deriving mapping algorithms to convert SF-36 scores into preference-based scoring systems such as EQ-5D, which can be presented as indices and are more useful for social analysis [24–26]. The results of our study indicate that, even in the general population, regular exposure to SHS at the workplace or at home has a negative effect on HRQOL measured using EQ-5D index/EQ-VAS score in a dose-dependent manner. Along with a previous study performed with a large scale cohort in Switzerland that showed that self-reported exposure to SHS was associated with lower HRQOL measured using SF-36, our results provide relevant data to help guide public health policies for the control of exposure to SHS in South Korea and other countries [12].

Although widely used, the EQ-5D has limitations that should be considered on interpretation. The EQ-5D is known to be relatively insensitive to change and has ceiling effects, especially in non-patient populations [27]. This might explain the relatively lower effect of exposure to SHS on changes in measures of EQ-5D index compared to other studies evaluating the SF-36 domains, and the discrepancy on the level of changes between EQ-5D index and EQ-VAS score, since EQ-VAS is more sensitive to change with fewer ceiling effects compared to EQ-5D index [12, 13, 28]. Nevertheless, the novel finding that exposure to SHS was independently associated with lower HRQOL measured with EQ-5D index/EQ-VAS score should not be underestimated.

Although the pathophysiological mechanisms of SHS exposure responsible for such detrimental effects on HRQOL remain unclear, there is indirect biological evidence to support our findings. In this respect, it is known that active smoking is associated with higher serum concentrations of epinephrine and norepinephrine and a lower concentration of renin, suggesting a potential interaction of exposure to smoke and the sympathetic regulatory system [29, 30]. Moreover, the increased exposure to chemical substances present in smoke, including nicotine and carbon monoxide, can cause peripheral vasoconstriction and impaired tissue oxygenation, leading to possible psychological derangement [14, 31]. Chronic inflammation caused by exposure to SHS, associated with increased concentrations of white blood cells, C-reactive proteins, fibrinogen, and various inflammatory cytokines, may also play a key role in the reduction in HRQOL [32]. Accordingly, long-term inflammatory changes due to repeated exposure to SHS may explain the dose-response relationship between exposure to SHS and reduced HRQOL shown in our study.

To interpret our results correctly, we should take into account the limitations of this study. First, collecting data on exposure to SHS by self-report may have led to recall bias or underestimation of the level of exposure, and potential misclassification with regard to true exposure, reflecting the perceived degree of exposure to SHS rather than the actual duration of exposure. However, previous studies have demonstrated a linear correlation between self-reported exposure to SHS and objective biomarkers such as serum cotinine, indicating that well-designed questionnaires can correctly estimate the actual relative levels of exposure to SHS, thereby supporting the validity of our results [33]. Second, because exposure to SHS of less than 1 hour per day was anchored to ‘0’ hours as responses to each question, the database could not provide accurate values as a continuous variable when calculating total exposure time in hours per day. Although disadvantages in analyzing such data exist from categorization, our results still provide relevant data on the dose-response effect. Third, as in any cohort study, there is the potential for selection bias, and confounding by unmeasured variables may have influenced the results.

The main strength of our study is the large sample size and the methodically randomized selection based on the survey data, which is representative of the general population of South Korea. In addition, by using a questionnaire to evaluate the smoking status and exposure to SHS of each respondent, we could effectively control for active smoking, which was a strong potential confounder in our analysis, and obtain information on the duration of exposure. Because the questionnaire used in the KNHANES V survey was based on various environmental factors, not only on exposure to SHS but also other potential hazards, possible recall bias was minimized.

In conclusion, daily exposure to SHS was associated with lower HRQOL measured using the EQ-5D index and EQ-VAS score in a dose-response manner. Importantly, these results will help guide policy recommendations on public health and tobacco control in South Korea and other countries.

## Supporting Information

S1 Table Multivariate Models of HRQOL Measures as a Function of Exposure to SHS (DOCX)

S2 Table Adjusted Differences in EQ-5D Index and EQ-VAS Score by Sex in the SHS-exposure Group Compared with the Unexposed Group* (DOCX)
