# Supplementary material for: Effect of Exposure to Second-Hand Smoke on the Quality of Life: A Nationwide Population-Based Study from South Korea
Source: PLoS One. 2015 Sep 22;10(9):e0138731. doi: 10.1371/journal.pone.0138731 (PMC4579139; doi:10.1371/journal.pone.0138731)
Supplement: S1 Table — (DOCX) [file pone.0138731.s001.docx]

**S1 Table . Multivariate Models of HRQOL Measures as a Function of Exposure to SHS**

| HRQOL measures | Variable Estimate (SE) | 95% CI | *P*-value |
| --- | --- | --- | --- |
| EQ-5D index |  |  |  |
| Exposure to SHS | -0.007 (0.003) | -0.013 - -0.002 | 0.005 |
| Age | -0.002 (0.000) | -0.002 - -0.002 | <0.001 |
| Sex | -0.018 (0.002) | -0.022 - -0.014 | <0.001 |
| BMI | -0.001 (0.000) | -0.001 - -0.000 | 0.043 |
| Education^*^ |  |  |  |
| Middle school | 0.040 (0.005) | 0.029 – 0.050 | <0.001 |
| High school | 0.046 (0.005) | 0.037 – 0.055 | <0.001 |
| Higher education | 0.041 (0.004) | 0.032 – 0.050 | <0.001 |
| Employed | 0.022 (0.002) | 0.017 – 0.027 | <0.001 |
| Individual economic status | 0.006 (0.001) | 0.004 – 0.009 | <0.001 |
| Any alcohol use | 0.009 (0.003) | 0.004 – 0.014 | 0.001 |
| Unmarried | -0.034 (0.003) | -0.040 - -0.027 | <0.001 |
| Any comorbid condition^†^ | -0.023 (0.003) | -0.029 - -0.018 | <0.001 |
| EQ-VAS score |  |  |  |
| Exposure to SHS | -1.936 (0.455) | -2.830 - -1.042 | <0.001 |
| Age | -0.169 (0.026) | -0.219 - -0.118 | <0.001 |
| Sex | -3.550 (0.517) | -4.567 - -2.534 | <0.001 |
| BMI | 0.003 (0.066) | -0.127 – 0.133 | 0.968 |
| Education |  |  |  |
| Middle school | 2.051 (0.873) | 0.336 – 3.765 | 0.019 |
| High school | 3.263 (0.780) | 1.730 – 4.795 | <0.001 |
| Higher education | 3.366 (0.799) | 1.797 – 4.936 | <0.001 |
| Employed | 1.603 (0.407) | 0.804 – 2.402 | <0.001 |
| Individual economic status | 1.250 (0.193) | 0.872 – 1.629 | <0.001 |
| Any alcohol use | 0.395 (0.484) | -0.556 – 1.345 | 0.415 |
| Unmarried | -4.267 (0.650) | -5.543- -2.990 | <0.001 |
| Any comorbid condition | -4.363 (0.515) | -5.374 - -3.352 | <0.001 |

HRQOL: health-related quality of life, SHS: second-hand smoke, CI: confidence interval

^*^Compared to elementary school or lower education

^†^Diabetes mellitus, hypertension, depression, stroke, osteoarthritis, asthma
