# Supplementary material for: Effect of Exposure to Second-Hand Smoke on the Quality of Life: A Nationwide Population-Based Study from South Korea
Source: PLoS One. 2015 Sep 22;10(9):e0138731. doi: 10.1371/journal.pone.0138731 (PMC4579139; doi:10.1371/journal.pone.0138731)
Supplement: S2 Table — (DOCX) [file pone.0138731.s002.docx]

**S2 Table. Adjusted Differences in EQ-5D Index and EQ-VAS Score by Sex in the SHS-exposure Group Compared with the Unexposed Group^*^**

| HRQOL measures | Women | | |  | Men | | | |
| --- | --- | --- | --- | --- | --- | --- | --- | --- |
|  | Variable Estimate (SE) | 95% CI | *P*-value | |  | Variable Estimate (SE) | 95% CI | *P*-value |
| EQ-5D index | -0.008 (0.003) | -0.014 - -0.002 | 0.014 | |  | -0.002 (0.003) | -0.008 – 0.005 | 0.628 |
| EQ-VAS score | -2.138 (0.525) | -3.169 - -1.107 | <0.001 | |  | -1.345 (0.890) | -3.093 – 0.403 | 0.131 |

SHS: second-hand smoke, HRQOL: health-related quality of life, CI: confidence interval

^*^Adjusted for age, body mass index, education, employed status, individual economic status, any alcohol use, marital status, and any comorbid condition (diabetes mellitus, hypertension, depression, stroke, osteoarthritis, asthma)
